# KUAKINI HHP CENTER FOR TRANSLATIONAL RESEARCH ON AGING: LATEST FINDINGS FROM MICE TO HUMANS

**DOI:** 10.1093/geroni/igac059.955

**Published:** 2022-12-20

**Authors:** Bradley Willcox, Richard Allsopp, Peter Martin

## Abstract

Kuakini Medical Center (Kuakini) was funded by NIH in late 2019 to create an interdisciplinary Hawai’i-based, Center of Biomedical Research Excellence (COBRE), for translational research on aging. This Center is building upon Kuakini’s five-decades of prior NIH-funded research. These resources include clinical data from the 57-year ongoing Kuakini Honolulu Heart Program (HHP), Honolulu-Asia Aging Study, HHP Offspring Study and a large biorepository. The Center’s overarching aim is to increase infrastructure for collaborative aging research in Hawaii. The first step is to grow the Center’s faculty by hiring and mentoring research project leaders (RPLs) from diverse disciplines to become independent, R01-funded, investigators on aging. Our first RPL has graduated after obtaining R01-funded status. His project utilizes novel CRISPR methods to i) improve the safety and efficacy of delivering potentially therapeutic genes (such as FOXO3) to the mouse genome, and ii) test whether temporal enhancement of FOXO3 expression improves healthy aging in this mouse model - both key steps for potential translation to human clinical therapies. This work will be highlighted in the Program Overview session followed by current RPL findings. The first of these current RPL talks presents data on a potential relation between the FOXO3 gene and vascular dementia in elderly Japanese-American males. The second talk reports on how strong social networks mitigate risk for dementia in elderly Japanese-American males. The third talk will report a relation between FOXO3-associated resilience to hypertension and lower intracerebral hemorrhagic stroke risk in elderly Japanese-American males. Supported by NIGMS 5P20GM125526 and NIA R01AG027060.

